# Novel Phenazine 5,10-Dioxides Release ^•^OH in Simulated Hypoxia and Induce Reduction of Tumour Volume *In Vivo*

**DOI:** 10.5402/2011/314209

**Published:** 2011-06-22

**Authors:** María L. Lavaggi, Mauricio Cabrera, Cristina Pintos, Carolina Arredondo, Gisela Pachón, Jorge Rodríguez, Stella Raymondo, José Pedro Pacheco, Marta Cascante, Claudio Olea-Azar, Adela López de Ceráin, Antonio Monge, Hugo Cerecetto, Mercedes González

**Affiliations:** ^1^Grupo de Química Medicinal, Laboratorio de Química Orgánica, Facultad de Ciencias-Facultad de Química, Universidad de la República, 11400 Montevideo, Uruguay; ^2^Cátedra de Análisis Clínicos, Laboratorio Central—Hospital Maciel (Ministerio de Salud Pública), Facultad de Química, Universidad de la República, 11200 Montevideo, Uruguay; ^3^Departamento de Patobiología, Facultad de Veterinaria, Universidad de la República, 11600 Montevideo, Uruguay; ^4^Department of Biochemistry and Molecular Biology, Faculty of Biology, Institute of Biomedicine of University of Barcelona (IBUB) and IDIBAPS, Unit Associated with CSIC, 08028 Barcelona, Spain; ^5^Departamento de Química Inorgánica y Analítica, Facultad de Ciencias Químicas y Farmacéuticas, Universidad de Chile, 83800005 Santiago, Chile; ^6^Centro de Investigaciones en Farmacobiología Aplicada, Universidad de Navarra, 31008 Pamplona, Spain

## Abstract

Phenazine 5,10-dioxides (PDOs) are a new class of bioreductive cytotoxins, which could act towards tumours containing hypoxic regions. The PDOs selective-hypoxic bioreduction was probed *in vitro*; however, the mechanism of action has not been completely explained. Besides, PDOs *in vivo* antitumour activities have not been demonstrated hitherto. We study the mechanism of hypoxic/normoxic cytotoxicity of PDO representative members. Electron spin resonance is used to confirm ^•^OH production, alkaline comet assay to determine genotoxicity, and gel electrophoresis and flow cytometry to analyze DNA fragmentation and cell cycle distribution. Chemically induced rat breast tumours are employed to evaluate *in vivo* activities. For the most selective cytotoxin, 7(8)-bromo-2-hydroxyphenazine 5,10-dioxide (PDO1), exclusive hypoxic ^•^OH production is evidenced, while for the unselective ones, ^•^OH is produced in both conditions (normoxia and simulated hypoxia). In normoxia (Caco-2 cells), PDO1 induces cell-cycle arrest and DNA fragmentation but does not significantly induce apoptosis neither at IC_50_ nor IC_80_. No difference in the comet-assay scores are observed in normoxia and simulated hypoxia being the unselective 2-amino-7(8)-bromophenazine 5,10-dioxide (PDO2) the most genotoxic. The *in vivo* efficacy with the absence of systemic toxicity of PDO1 and PDO2 is checked out. Results from this study highlight the potential of PDOs as new therapeutics for cancer.

## 1. Introduction

**Figure 1:**
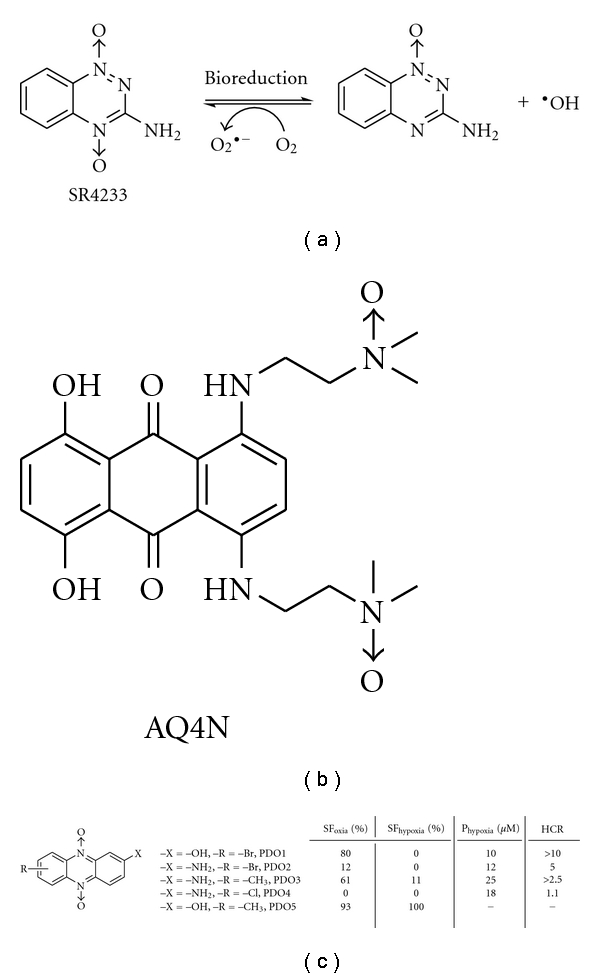
(a) The chemical structure and proposed mechanism of action of SR4233. (b) The chemical structure of AQ4N. (c) The chemical structures and *in vitro* biological behaviour of PDOs studied here. SF: survival fraction of V-79 cells treated with PDOs at 20 *μ*M; P_hypox_: Hypoxic potency, dose which gives 1% of control cell survival in hypoxia; HCR: dose in air divided by the dose in hypoxia giving 1% of control cell survival.

Tumours are heterogeneous and contain hypoxic and anoxic regions, which alter cellular metabolism tending to select for a more malignant phenotype, which increases mutation rates, increases expression of genes associated with angiogenesis and tumour invasion, and is associated with a more metastatic phenotype of human cancers [1, 2]. By enhancing metastasis, hypoxia compromises curability of tumours by surgery. Conversely, the hypoxic cells are associated with increased resistance to radiation and chemotherapy [3]. Due to the inadequate vascularisation of solid tumours, drugs do not reach hypoxic cells in adequate concentrations. Also, conventional anticancer drugs in clinical use are antiproliferative agents that kill dividing cells, by attacking DNA (synthesis, replication, or processing), being ineffective in the hypoxic tumours, because these cells are not dividing rapidly. However, hypoxia has been identified as an important tool for the specific activation of some antitumour prodrugs, namely, bioreductive agents [4]. These prodrugs are inactive in well-oxygenated tissues and are selectively biotransformed to active cytotoxic species in hypoxic cells. One of the most studied bioreductive agent is the *N-*oxide, tirapazamine (SR4233, Figure 1(a)) [5, 6] which after one-electron reductive activation releases hydroxyl free radical (^•^OH, Figure 1(a)) producing oxidative DNA damage without covalent binding to DNA and proteins [7]. Additionally, hybrid compounds that combine an *N*-oxide and a ¶-DNA stacking moieties have been described as enhanced-cytotoxic agents (AQ4N, Figure 1(b)) [8]. In this sense, we have designed a series of phenazine 5,10-dioxides (PDOs) as prodrugs that could damage tumour hypoxic cells through bioreduction, generating free radicals and DNA damage by intercalation. Some of the developed PDOs are *in vitro* hypoxic selective cytotoxins, that is, PDO1 (Figure 1(c)), others are hypoxic partially—or nonselective cytotoxins, that is, PDO2, PDO3, and PDO4, respectively (Figure 1(c)), and others are noncytotoxic, that is, PDO5 (Figure 1(c)) [9–12]. Besides, some of the PDOs displayed *in vitro* aerobic-antiproliferative activity against Caco-2 cells [13]. The selective hypoxic reduction and its relationship to bioreductive activity were proved using enzymatic mammal systems [14]. 

To get insight into the mechanism of PDOs hypoxic/oxic cytotoxicity, herein, we depict a series of experiments. Electron spin resonance (ESR) experiments, in aerobic—and anaerobic—metabolic conditions, were done to confirm the hypothesis that hypoxic-selective PDOs could selectively produce ^•^OH in system with low oxygen content like SR4233 (Figure 1(a)). Additionally, PDOs oxidative genetic damage was evaluated by the modified comet assay after postdigestion of the cells with formamidopyrimidine-DNA-glycosylase (FPG) and endonuclease III (EIII). For the best selective derivative, PDO1, aerobic-cytotoxicity mechanism on human colorectal adenocarcinoma cell line was studied analyzing the cell growth inhibition capacity, by MTT assay, the capability to alter the cell cycle and the possibility to induce apoptosis, by fluorescence-activated cell sorter (FACS) and Hoescht analysis, and the capacity to produce DNA fragmentation by agarose gel electrophoresis. Due to the ESR experiments showed that some PDOs were able to produce ^•^OH in normoxia, we also studied the effect of vitamin C (vitC) on the bioreductive profiles. This study [15] tried to determine if this antioxidant modifies PDOs biological behaviours, especially exhibiting a protective action in normoxic cells. To select the type of tumour for the *in vivo* studies, bioreductive profiles against a set of tumour cells (MCF-7, TK-10, and HT-29) were determined. From these results, the PDOs were studied *in vivo* on a chemically induced model of rat breast tumours, evaluating initially the systemic toxicity of compounds on healthy animals. The *in vivo* antitumour efficacies were evaluated analysing tumours volumes and histopathologies.

## 2. Materials and Methods

### 2.1. Reagents and Cell Culture

PDO1-PDO5 were prepared according to previous description [9]. All the reagents and solvents for syntheses and ESR experiments were purchased from Sigma-Aldrich (St. Louis, Mo, USA). V79 cells (Chinese hamster lung fibroblasts) were obtained from European Collection of Animal Cell Cultures, and maintained in logarithmic growth as subconfluent monolayer by trypsinisation and subculture to (1-2) × 10^4^ cells/cm^2^ twice weekly. The growth media was Eagle's Minimal Essential Medium (EMEM, Gibco, Prat de Llobregat, Barcelona, Spain), containing 10% (v/v) foetal bovine serum (FBS, Gibco, Prat de Llobregat, Barcelona, Spain) and penicillin/streptomycin at 100 U/100 *μ*g/mL. Caco-2 cells (ATCC/HTB-37, ATCC, Manassas, VA, USA), were grown in Dulbecco's Modified Eagle's Medium (DMEM, Gibco, Prat de Llobregat, Barcelona, Spain) supplemented with 10% FBS and 1% antibiotic (10,000 U/mL penicillin and 10,000 Ug/mL streptomycin, Gibco, Prat de Llobregat, Barcelona, Spain). Cells were maintained as monolayer cultures at 37°C in a humidified atmosphere with 5% CO_2_. An adequate number of MCF-7 (human mammary adenocarcinoma, ATCC HTB-38), TK-10 (human kidney carcinoma, NCI), and HT-29 (human colon adenocarcinoma, ATCC HTB-38) cells, were maintained in RPMI-1640 growth media, supplemented with L-glutamine (1%), penicillin/streptomycin (1%), nonessential amino acids (1%), and 10% (v/v) FBS. The cultures were maintained at 37°C and 5% CO_2_ for 48 h. The absorbance at 540 nm before the treatment was determined.

### 2.2. Free Radical Measurements

All ESR experiments were conducted using a Bruker ECS 106 spectrometer (Bruker Instruments Inc., Billerica, Mass, USA) in the X band (9.85 GHz) using a rectangular cavity and 50 kHz field modulation. All the spectra and the hyperfine couplings (to 0.1 G) were registered in the same scale and after 15 scans.

#### 2.2.1. Preparation of the Rat Liver Cytosolic Proteins

Livers were obtained from female Wistar rats (250–300 g), provided by the “Centro de Investigaciones Nucleares”, UdelaR (Montevideo, Uruguay). The animals were allowed food and water *ad libitum*. The experimental protocols with animals were evaluated and supervised by the local Ethics Committee and the research adhered to the Principles of Laboratory Animal Care [16]. The animals were sacrificed by cervical dislocation and the livers, maintained in a ice bath, were perfused in situ with an ice-cold NaCl (0.9%) solution and washed with 3 volumes of Tris-HCl (0.05 M)-sucrose (0.25 M) pH = 7.4, then they were sliced and homogenised in a Potter-Elvehjem glass-Teflon homogeniser. The homogenates were centrifuged for 30 min at 900 ×g at 4°C and the supernatant fraction was centrifuged at 10,000 ×g for 1 h at 4°C. The pellet was discarded and the supernatant fraction was further centrifuged at 100,000 ×g for 1 h at 4°C. The cytosolic fraction, supernatant, was recovered. Protein content was determined by the bicinchoninic acid assay from Sigma (St. Louis, MO, USA) as suggested by the manufacturer. 

#### 2.2.2. Radical Production Measuring

PDO1-PDO3 or PDO5 (1 mM in DMSO), the spin trap *N-tert*-butyl-*α*-(4-pyridyl) nitrone *N′*-oxide (POBN, 100 mM), and rat liver cytosolic proteins (1 mg/mL) in phosphate buffer (0.1 M, 1.5 mM EDTA, pH = 7.4) were mixed at 37°C in the ESR cell and gassed with nitrogen (simulated-hypoxia) or oxygen (simulated oxia) for 20 min. After that time, the NADPH was added. The spectra were recorded after 15 scans. The control incubations, in both conditions, were without NADPH-generating system.

### 2.3. Alkaline Comet Assay

Monolayer V79 cells in exponential growths were trypsinised (Trypsin, Gibco, Prat de Llobregat, Barcelona, Spain) and suspension cultures were prepared in 50 mL glass flasks: 3.3 × 10^4^ cells/mL in 30 mL of growth media. The glass flasks were topped with rubber caps perforated with two 21 G needles (Microlance, Becton Dickinson, Fraga, Huesca, Spain) to provide gas inlet and outlet in order to generate hypoxia and well-oxygenated conditions. They were placed on a shaking device introduced in a water bath at 37°C and were gassed with humidified air (oxygenated experiment) or with nitrogen (hypoxic experiment) during all the experiment. Different concentrations of PDO1 and PDO2 in 100% dimethylsulfoxide (DMSO, Panreac, Montcada i Reixac, Barcelona, Spain) were prepared just before dosing. After 30 min of gassing, 200 *μ*L of each solution were added to reach the following final concentrations: 10 and 20 *μ*M in oxygenated conditions and 1, 5, and 10 *μ*M in hypoxia. At these concentrations, the viability is higher than 90%. A control with the solvent was included under both conditions. After 2 h of treatment, cells were centrifuged at 175 × g and resuspended in 1 mL of growth media. The cell concentration was adjusted to 6.25 × 10^5^ cells/mL in PBS. Forty microliters were mixed with 130 *μ*L of 1% low-melting-point agarose (Sigma-Aldrich, St. Louis, Mo, USA) and 80 *μ*L was spread onto microscope slides (Menzel-Glaser, Braunschweig, Germany) precoated with 0.5% of normal-melting-point agarose (Sigma-Aldrich, St. Louis, Mo, USA). Three slides were prepared for each condition, slides 1, 2, and 3. Glass cover slips (Menzel-Glaser, Braunschweig, Germany) were placed on the gels, which were allowed to set at 4°C. Then, the cover slip was removed and the cells embedded in agarose were lysed for 1 h by immersion of the slides in 2.5 M NaCl, 100 mM Na_2_–EDTA, 10 mM Trizma–HCl, pH = 10 and 1% Triton X-100 at 4°C. After that the slides were washed three times (5 min each time) with enzyme buffer (0.1 M KCl, 0.5 mM Na_2_–EDTA, 40 mM HEPES-KOH, 0.2 mg/mL BSA, pH = 8.0) and incubated for 45 min at 37°C with FPG in the enzyme buffer—slide 1, or EIII in the enzyme buffer—slide 2, or with buffer alone—slide 3. Then, the slides were placed on a horizontal gel electrophoresis tank and the DNA was allowed to unwind for 40 min in freshly prepared alkaline electrophoresis buffer (300 mM NaOH and 1 mM Na_2_–EDTA, pH > 13). Electrophoresis was run in the same buffer for 30 min at 25 V (about 0.8 V/cm across the gels and approximately 300 mA) in an ice bath condition. The slides were rinsed three times (5 min each time) with 400 mM Trizma (pH = 7.5) to neutralize the excess alkali. Then, the slides were washed in water and drained overnight. Gels were stained with 25 *μ*L of 1 *μ*g/mL DAPI (Sigma–Aldrich, St. Louis, Mo, USA), covered with a cover slip and coded before microscopic analysis. DAPI stained nuclei were evaluated with a Nikon Eclipse TE 300 fluorescence microscope (Nikon, Tokyo, Japan). A total of 100 comets on each gel were visually scored and classified as belonging to one of five classes according to the tail intensity. Each comet class was given a value between 0 and 4: (0) = no damage and (4) = maximum damage. The total comet score (tcs) was calculated by the following equation: (percentage of cells in class 0 × 0) + (percentage of cells in class 1 × 1) + (percentage of cells in class 2 × 2) + (percentage of cells in class 3 × 3) + (percentage of cells in class 4 × 4). Consequently, the tcs was in the range from 0 to 400. Controls were included, only solvent for negative and treatment with hydrogen peroxide (50 *μ*M) during 5 min on ice for positive one. Experiments were performed in triplicate.

### 2.4. Oxic-Studies on Caco-2 Cells

#### 2.4.1. Antiproliferative Activity

The assay was performed using previously described method slightly modified [17]. Samples containing 200 *μ*L cell suspension (2 × 10^4^ cells/mL) were plated in 96-well-flat-bottomed microtiter plates. After adherence of the cells within 24 h of incubation at 37°C, PDO1 and PDO2 at doses ranging from 1 *μ*M to 1000 *μ*M were added to different wells (3-4 per concentration). After additional incubation time (24, 48, and 72 h) at 37°C in a humidified incubator with 5% CO_2_, MTT dissolved in PBS and sterile filtered was added to all the wells at a final concentration of 1 mg/mL. Following 1 h of incubation, the generated formazan was dissolved with 100 *μ*L DMSO per well. The optical density was measured using an ELISA plate reader (Merck ELISA system MIOS version 3.2.) at 550 nm. The concentrations that caused 50 and 80% inhibition of cell growth (IC_50_ and IC_80_) were calculated.

#### 2.4.2. Cell Cycle Analysis

Cell cycle was assessed through flow cytometry by using a FACS. Cells were cultured in 6-well flat bottomed microtiter plates containing 2 mL of cell suspension. The number of cells was determined by calculation according to the number of cells/wells in 96-well plates (582,000 cells). After 24 h of incubation at 37°C with 5% CO_2_, PDO1 was added at IC_50_ dose. Following 24 h of incubation, cells were harvested by mild trypsinisation, collected by centrifugation and stained in Tris buffered saline for 1 h at 4°C. FACS analysis was carried out at 488 nm in an Epics XL flow cytometry (Coulter Corporation, Hialeah, Fla, USA). Data from 12,000 cells were collected and analysed using Multicycle program (Phoenix Flow Systems, San Diego, Calif, USA). All experiments were performed in triplicate and repeated three times.

#### 2.4.3. Assessment of Apoptosis

Apoptosis was assessed using Annexin V-fluorescein isothiocyanate (FITC) kit binding assay and analyzed by FACS. Cell culture and treatment with PDO1 was carried out as described in cell-cycle analysis section. Thereafter, cells were resuspended in binding buffer (10 mM Hepes/NaOH, pH = 7.4, 140 mM NaCl, 2.5 mM CaCl_2_). Annexin V-FITC (Bender System Kit) was added according to the product insert and incubated for 30 min at room temperature in the dark. One min before FACS analysis, propidium iodide (PI) was added at a concentration of 20 *μ*g/mL. Approximately 500.000 viable cells were counted to assess apoptosis. Experiments were performed in triplicate.

#### 2.4.4. Apoptosis Determination with Bisbenzimide Hoechst, to Evaluate Chromatin Condensation

Apoptosis was assessed using bisbenzimide Hoechst and analyzed by Fluorescence-Microscope. Cells were cultured in 6-well flat bottomed microtiter plates containing 2 mL of cell suspension. The number of cells was determined by calculation according to the number of cells/wells in 96-well plates (12,000). After 24 h of incubation at 37°C with 5% CO_2_, PDO1 was added at its respective IC_50_ and IC_80_ doses. Following 24 h of incubation in the absence or presence of the indicated compound, cells were harvested by mild trypsinisation, collected by centrifugation and fixed with paraformaldehyde at 3.7% for 10 min at −20°C. Cells were washed with PBS, centrifuged at 2500 rpm for 5 min, Triton-X100 at 0.5% was added for 5 min at 4°C and cells were stained with 50 ng/mL Hoechst 33258 dye for 15 min and then placed onto slides, coverslips were mounted with Mowiol 4–88. Chromatin condensation was visualized by fluorescence microscopy.

#### 2.4.5. DNA Fragmentation Study

DNA fragmentation was determined using “Real Pure Extraction Kit”. In short, 2 × 10^6^ cells/well were cultured in 100-mm plates and treated for 24 h with PDO1 at IC_50_ dose. Chromosomal DNA was isolated (Real, Durviz, Valencia, Spain) and ladder formation was analysed in a 1% agarose gel visualizing with ethidium bromide staining. Staurosporine (10 *μ*M) was used as positive control. This assay was performed on four separate occasions for statistical analysis [18].

### 2.5. Clonogenic Bioreductive Assays

Monolayers of cells (V79, MCF-7, TK-10, or HT-29) in exponential growth were trypsinised, and suspension cultures were set up in 50 mL glass flasks: 2 × 10^4^ cells/mL in 30 mL of the corresponding growth media. The glass flasks were submerged and stirred in a water bath at 37°C, where they were gassed with humidified air or pure nitrogen. Treatment [19]: compounds solutions were prepared just before dosing. Stock solutions, 150-fold more concentrated, were prepared in pure DMSO (Aldrich, St. Louis, Mo, USA) or sterilized distilled water. Thirty min after the start of gassing, 0.2 mL of the stock compound solution was added to each flask, two flasks per dose. In every assay, there was one flask with 0.2 mL of DMSO (negative control). In the assays with vitC, after 5 min of gassing the cells suspension, with air or nitrogen, 100 *μ*L of vitamin solution was added to reach the same concentrations that the studied PDOs and 25 min pretreatment before compounds addition was done. Cloning: after 2 h exposure to the compound, the cells were centrifuged and resuspended in plating medium. Cell numbers were determined with a haemocytometer and 10^2^–10^3^ cells were plated in 6-well plates to give a final volume of 2 mL/30 mm of well. Plates were incubated at 37°C in 5% CO_2_ during 7 days, for V79, or 14 days, for MCF-7, TK-10, and HT-29, and then stained with aqueous crystal violet. Colonies with more than 64 cells were counted. The plating efficiency (PE) was calculated by dividing the number of colonies by the number of cells seeded. The percent of control-cell survival for the compound-treated cultures (SFnormoxia and SFhypoxia) was calculated as PE-treated/PE-control 100. The compounds were tested at different doses in duplicate flasks both in normoxic and hypoxic conditions.

### 2.6. *In Vivo* Studies

#### 2.6.1. Animals

Specific pathogen-free 180-day-old adult (for maximum tolerated doses (MTD) studies and toxicity in healthy animals studies) or 60-day-old adult (for *in vivo* tumour studies) female Sprague-Dawley rats were purchased from Centro de Investigaciones Nucleares-Universidad de la República (Montevideo, Uruguay). Rats were housed in sterile individually ventilated cages; food and water were provided *ad libitum*, in accordance with the standard operating procedures set down in SI 17/94 of the European Union. Rats were acclimatised for 1 week before the initiation of any *in vivo* experiments. Animals were sacrificed by cervical dislocation if found to be in distress (hunching, failure to groom, etc.) or if tumour volume exceeded 10% of mouse bodyweight. All experiments were performed according to the Local Animal Ethics Committee (Universidad de la República Ethical Committee, Montevideo, Uruguay) guidelines for animal experimentation.

#### 2.6.2. Formulation of Compounds for *In Vivo* Trials

PDO1 and PDO2 were suspended in sterile saline: Tween80 (4 : 1) (vehicle solution) immediately prior to injection. These preparations were made under aseptic conditions and in all cases suspensions were obtained by shaking under ultrasound conditions.

#### 2.6.3. *In Vivo* Dose Treatment Determination

In order to determine the *in vivo* dose with the minimum associated-toxicity first at all the MTDs (dose giving 20% weight loss within 3 days of a single ip administration) for PDO1 and PDO2 were determined [19]. Consequently, three nontumour-bearing animals per dose level were injected intraperitoneally with a single dose of compound, at 60, 120, or 300 mg/kgb.w. Animals were weighed and observed daily for alterations in skin, physical aspect, activity and faeces aspect. After that, nontumour-bearing animals were treated intraperitoneally, at a dose equivalent to MTD/6, according to schedule showed in Figure 1S (see Appendix. Supplementary data, which is located at doi: 10.5402/2011/314209). Animals were sacrificed at the experimental end point (Figure 1S), the organs (lung, kidney, liver, spleen, heart, and intestine, maintained in aqueous formalin solution (10%)) for further histological studies were obtained by autopsy and blood for biochemical and haematological studies was drawn by sectioning the subclavian artery. Biochemical and haematological determinations were done immediately or no more than 24 h post-extraction maintaining the blood in EDTA or heparin at 0°C.

#### 2.6.4. Tumour Model: Chemically Induced Rat Mammary Tumours

Twenty-twenty five rats were treated with 5 mg/kgb.w. of *N-*nitrosomethylurea (NMU, Sigma-Aldrich, St. Louis, MO, USA, dissolved in acidified saline) by tail-vein injection at 60, 90, and 120 days of age [20]. Animals were palpated twice a week 60 days after the last NMU administration in order to record the presence, location, size, and date of detection for all tumours. Induction efficiency of 50% was obtained.

#### 2.6.5. *In Vivo* Antitumour Studies

When the tumours reached approximately 4 mm in diameter the rats were randomly divided into three groups (untreated animals, PDO1-, or PDO2-treated). The treatment groups received an ip injection of PDOs, at a dose equivalent to MTD/6; once daily according to schedule showed in Figure 1S (see Appendix. Supplementary data). Control rats received in the same schedule only vehicle solution. Tumour growth was measured with a sterile calliper. The long (L) and short (S) axes were recorded, and tumour volume (V) was calculated using the following equation as described previously [21]:

(1) $$V=\frac{\left(S^{2}\times L\right)}{2}.$$

Regarding statistical analysis, Student's *t*-test was conducted in a comparison between two groups.

Animals were sacrificed at the experimental end point (Figure 1S), the organs (lung, kidney, liver, spleen, heart, and intestine, maintained in aqueous formalin solution (10%)) and tumours for further histological studies were obtained by autopsy and blood for biochemical and haematological studies was drawn by sectioning the subclavian artery. Biochemical and haematological determinations were done immediately or no more than 24 h after extraction maintaining the blood in EDTA or heparin at 0°C. Sections from organs and tumours were fixed in 10% neutral buffered formalin and embedded in paraffin. Blocks were sectioned and fixed on slides to be evaluated histopathologically.

## 3. Results and Discussion

**Figure 2:**
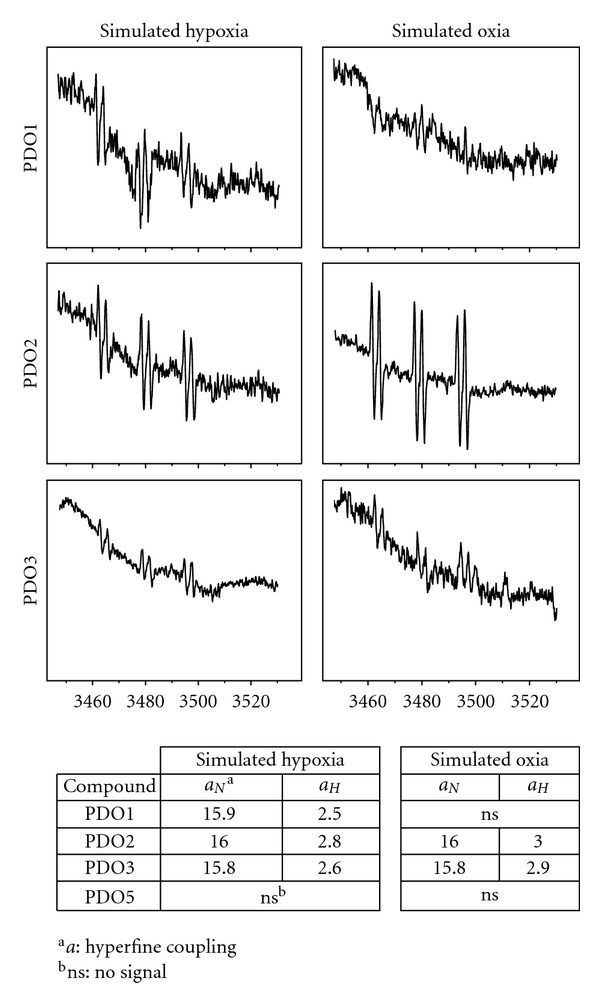
ESR studies of PDOs in bioreductive system (rat liver cytosolic proteins, 1 mg/mL, +NADPH). ESR spectra of PDOs (1 mM, treated as indicated in Material and methods section) were recorded in presence of the spin trap POBN (100 mM). Number of scans: 15.

In order to probe that the PDO bioreduction is associated to ^•^OH production and to know whether it occurs according to compounds selectivity profiles, some PDOs were metabolised under normoxia and simulated hypoxia and ESR spectra were acquired. Therefore, one selective, PDO1, two partially selective, PDO2 and PDO3, and one noncytotoxic derivative, PDO5 (Figure 1(c)), were included in this study. Except for compounds PDO1 in normoxia and PDO5 in both conditions the spectra show a double triplet signal (Figure 2) with hyperfine splitting of *a*_*H*_ = 2.9 and *a*_*N*_ = 16.0 G, which pattern is attributed of the spin trap(POBN)-^•^OH adduct. Spin trap-ESR experiments clearly demonstrated PDOs were able to release ^•^OH in presence of bioreductive systems finding that non selective and cytotoxic derivative, that is, PDO2, produces it under both conditions contrary non active derivative, that is, PDO5, shows no free radical production (no signal in the ESR spectrum). While the *in vitro* hypoxic selective cytotoxin PDO1 releases ^•^OH only under simulated hypoxia. For PDO2 and PDO3 in normoxia the identified ^•^OH could be the result of a “futile redox cycling”, where the produced superoxide radical, O_2_^•−^, via spontaneous dismutation gives H_2_O_2_ that could be reduced to the ^•^OH free radical. These results confirm that cytotoxic selectivity could be due to the selective radical species generation during the bioreduction process under hypoxic conditions as it is described for SR4233 [7].

**Figure 3:**
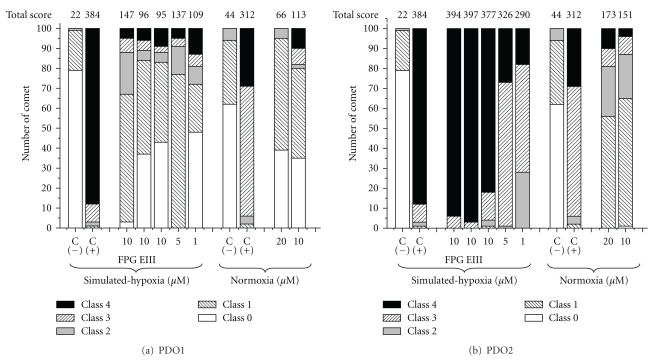
Alkaline comet assay analysis on V79 cells incubated with the studied compounds in different conditions and doses, expressed as cells in five different classes (0–5) and quantified as Collins et al. [22]. C(−): negative control (only solvent); C(+): positive control (treatment with hydrogen peroxide, 50 *μ*M, during 5 min on ice).

To evaluate the role of ^•^OH on the DNA damage we checked, using alkaline comet assay, whether PDO1 and PDO2 were able to generate DNA strand breaks and oxidative DNA damage. This last phenomenon could be probed by alkaline comet assay using two enzymes [23], that is, FPG and EIII. FPG is involved in the first step of base excision repair to remove specific modified bases from DNA excising mainly 2,6-diamino-4-hydroxy-5-*N*-methylformamidopyrimidine and 8-oxo-G. EIII nicks DNA at sites of oxidised-pyrimidines.  Figure 3 shows the comet assay results obtained with PDO1 and PDO2 in the different experimental conditions. Under simulated-hypoxia the treatment with PDO1 during 2 h (Figure 3(a)) increased moderately the total comet score (tcs) of V79 cells comparing to hypoxic untreated cells (C(−), Figure 3(a)) however lower than the tcs of positive control (C(+): H_2_O_2_, 50 *μ*M, during 5 min on ice) at all the studied doses. Additionally, PDO1 was less genotoxic in both conditions than previous studied quinoxaline dioxide [15]. No dose dependence was observed either in simulated hypoxia or in normoxia. Interestingly, according to a significant increase in the tcs when the V79 cells were digested with FPG, PDO1 could produce genotoxicity in hypoxia by production of 8-oxo-G. No differences were found between treatments with and without EIII showing absence of hypoxic-PDO1 oxidizing capability on pyrimidines. On the other hand, PDO2 produced (Figure 3(b)), in a dose-dependent manner and in both conditions, higher tcs than negative control and PDO1 being the results more notorious in simulated hypoxic conditions. There were differences between the hypoxic tcs when the digestions were done with both FPG and EIII and without them showing PDO2 hypoxic genotoxicity could be the result not only of the production of oxidised-purines (8-oxo-G) but also oxidised pyrimidines. These results indicate PDO1 and PDO2 promote in hypoxia DNA-oxidative damage yielding oxidised purines or oxidised purines and pyrimidines, respectively, like SR4233. In agreement with the ESR experiments in normoxia, PDO2 produced higher DNA-strand breaks than PDO1.

For the best selective derivative, that is, PDO1 (better *in vitro *P_hypox_ and HCR, Figure 1(c)), aerobic-cytotoxicity mechanism on human colorectal adenocarcinoma cell line, Caco-2, was studied. According to our previous results PDO1 showed low oxic cytotoxicity [9], and this, according to results herein depicted, could be related to DNA damage (Figure 3(a)) and not to ^•^OH production (Figure 2).

Caco-2 growth inhibition capacity of derivative PDO1 was studied by MTT assay, its capability to alter the cell cycle and the possibility to induce apoptosis were examined using fluorescence-activated cell sorter (FACS) and Hoescht analysis, and its capacity to produce oxic DNA fragmentation was analyzed by agarose gel electrophoresis.

**Figure 4:**
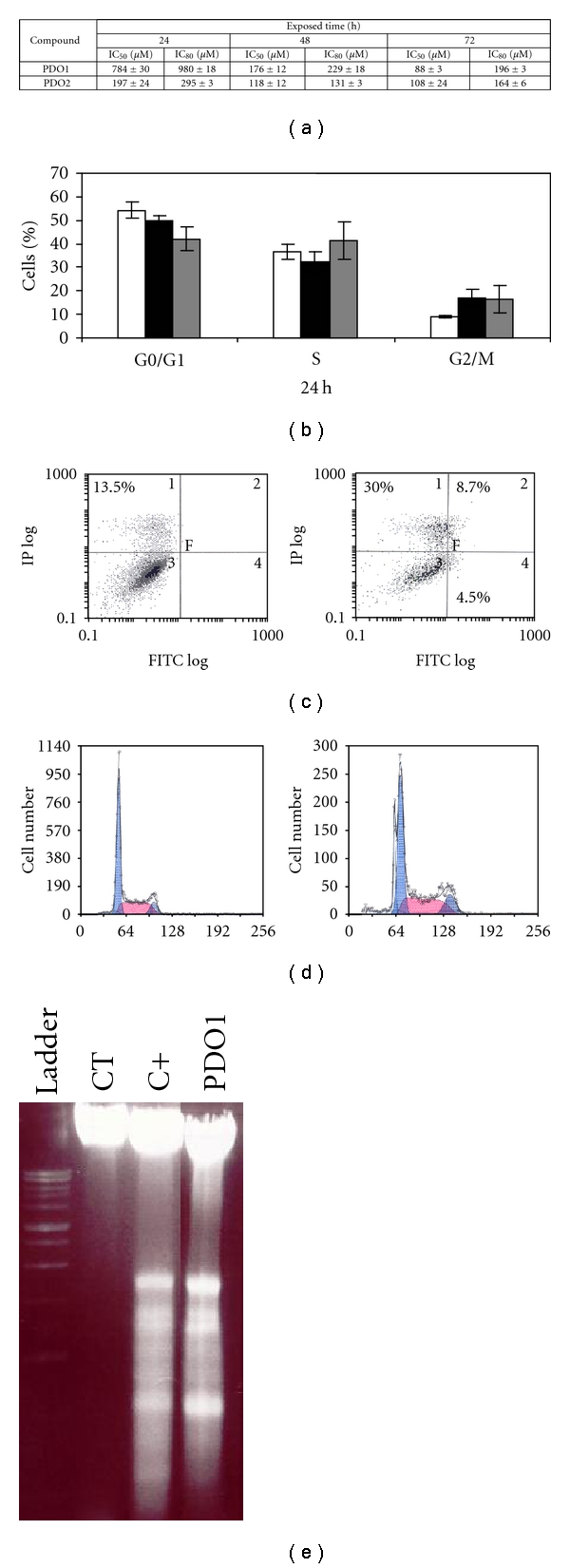
(a) IC_50_ and IC_80_ of PDO1 and PDO2 on Caco-2 cells after 24, 48, and 72 h incubation. (b) Percentage of distribution in different phases of cell cycle of untreated (□) and treated Caco-2 cells after 24 h with PDO1 at respective IC_50_ (■) and IC_80_ (■). (c) Percentage of early and late apoptotic and necrotic cells assessed by flow cytometry analysis of Annexin V-FITC staining and PI accumulation after exposure of Caco-2 cells to PDO1 at respective IC_50_ and IC_80_ after 24 h of incubation. (d) Apoptotic peaks detected by flow cytometry in treated Caco-2 cells after 24 h incubation with untreated (left) and PDO1-treated (right) at respective IC_80_. (e) Agarose gel electrophoresis analysis of DNA from Caco-2. Cells were incubated in the absence of substances (CT) or treated with PDO1 at IC_50_ or staurosporine (10 *μ*M) (C+) for 24 h.

As indicated by the IC_80_, PDO1 was less Caco-2-antiproliferative agent than PDO2 in oxic conditions (Figure 4(a)). PDO1 was able to induce an arrest in G2/M cell cycle phase, after 24 h and at IC_50_ dose (Figure 4(b)), which led cells to necrosis confirmed by Annexin V analysis. However, PDO1 also produced apoptotic cells at IC_50_ and IC_80_ doses, confirmed by Hoechst (data not shown) and by the appearance of DNA-ladder pattern (Figures 4(c)–4(e)). These experiments highlight the relevance of PDO1 as potential drug in overcoming cancer.

Due to the ESR experiments showed that PDO2 and PDO3 were able to produce ^•^OH in normoxia, we studied the effect of vitC on their V79-hypoxic and normoxic cytotoxicities. Therefore, to see whether vitC had protective activity in oxygenated systems and prooxidant activity in hypoxic ones we performed an *in vitro *bioreductive study preincubating the cells with vitC 25 min before the incorporation of PDO2, PDO3, or PDO4 into to the milieu. PDO3 and PDO4 selective cytotoxic profiles were modestly modified to most selective behaviours in presence of vitC (Table 1) which apparently scavenges reactive oxygen radicals in normoxia. However, in hypoxic conditions, and also for PDO2, vitC potentiates compounds cytotoxicity in V79 cells [24], like it was described previously for SR4233 [25]. This information could be used for future cancer treatment schedules.

To select the type of tumour for *in vivo* studies, hypoxic and normoxic cytotoxicity against a set of tumour cells, MCF-7, TK-10, and HT-29, were performed with the hypoxic partially selective cytotoxins PDO2. From these studies (Table 1), it could be seen partially selective cytotoxic profiles on the three studied tumour cells, like in V79 ones, showing the major cytotoxicity against human breast cancer cells (MCF-7).

After the demonstration that hypoxic selective PDO1 induce some degree of apoptosis in oxia and hypoxic non-selective PDO2 is not just specific for any of the studied tumour cells but awfully toxic for MCF-7, we next sought to determine the effects of both compounds in an *in vivo* breast-cancer model. We selected the model of NMU chemically induced breast tumours because these tumours have hypoxic regions [26], and consequently, it is appropriate to study the *in vivo* behaviour of hypoxic cytotoxin PDO1.

Initially, we confirmed that PDO1 and PDO2 did not adversely affect healthy animals being the MTD higher than 300 mg/kgb·w. (Table 1S, Appendix. Supplementary data). Higher doses were not assayed due to solubility problems. Both derivatives have very good values of MTD in comparison to values in mice for similar dioxides (quinoxalines and SR4233) [19, 27]. Also, four-week treatments were performed according to the selected schedule (Figure 1S, Appendix. Supplementary data) using a dose of 50 mg/kgb·w/day equivalent to MTD/6. In this condition, animals showed no alteration of behaviour during the study and the mortalities were 0%. Organs histology (data not shown) and biochemical and haematological findings (Table 2S, Appendix. Supplementary data) showed this treatment did not affect the healthy and strength of the animals.

**Figure 5:**
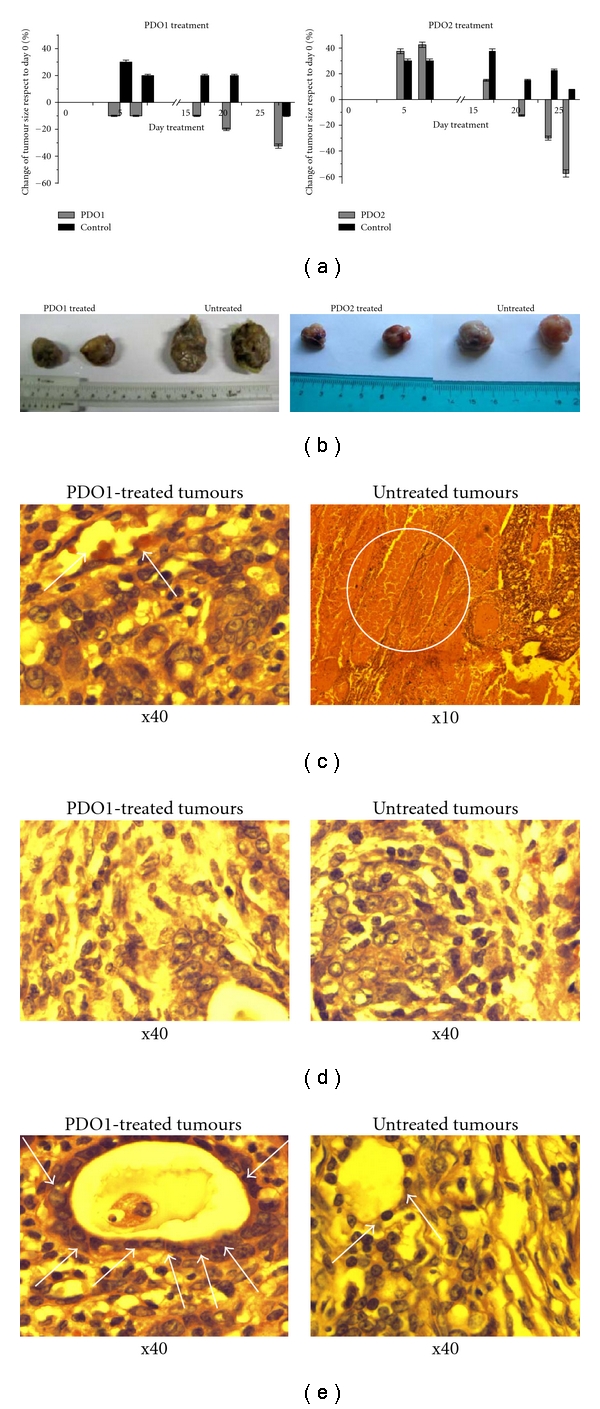
Antitumour effects of PDO1 and PDO2. (a) Tumour growth was measured every week and the change in the size was expressed respect to day 0 (beginning of the treatment). Results are expressed as the mean (*n* = 4 per group, statistical analysis was performed using the Student's unpaired *t*-test, **P < .01). Rats were killed on day 29, organs and (b) tumours were removed comparing size and weight of treated and untreated. Histopathology findings (haematoxylin/eosin slides) of PDO1-treated (left) and untreated (right) breast NMU-induced tumours demonstrated: (c) absence of necrosis and vascularisation (arrows) for PDO1 treated (left) and necrosis (circle) for untreated; (d) different tumour compartmentalisation behaviours (presence, left, and absence, right) and micrometastases for untreated (right); (e) differential infiltration of lymphocytes and eosinophils (arrows), significant immune response for PDO1-treated (left) and irrelevant for untreated (left).

Secondly, treatments with PDO1 and PDO2, according to schedule shown in Figure 1S (Appendix. Supplementary data), to breast-tumour-bearing animals resulted in strong and significant decreases in tumour size indicating that both PDO1 and PDO2 significantly impeded tumour growth (Figure 5(a)). However, in untreated animals, the tumour grew progressively. Whereas for PDO2 the size tumours diminution, respect to day 0, was evident at day 20th for PDO1 was manifested at the treatment's beginning. During PDO1 and PDO2 treatments no alteration of the animals was observed and mortality was 0%. From the necropsies, at experimental end point day 29th, were obtained the tumours (Figure 5(b)) and organs. The histopathologic studies of PDO1-treated tumours showed absence of necrotic zones and good vascularisation (arrows in Figure 5(c)), while the untreated tumours had full necrosis areas (circle in Figure 5(c)). In addition, the size reduction could be the result of the tumour compartmentalisation as result of the collagen from the tumour capside (Figure 5(d)). This compartmentalisation could produce tumour packing and further fragmentation. Moreover, the untreated tumours showed local metastasis and absence of compartmentalisation phenomenon (Figure 5(d)). Additionally, in the PDO1-treated tumours a desmoplastic and peritumour reaction was observed with infiltration of lymphocytes and eosinophils (arrows in Figure 5(e)). This high infiltration could be indicating an enhanced of the immune response against the tumour. The untreated tumour showed a lower infiltration (arrows in Figure 5(e)). Table 3S (Appendix. Supplementary data) summarises these observations.

In conclusion, we completed the information related to PDOs mechanism of cytotoxicity and *in vivo* behaviours. The PDOs acted as bioreductive agents by hypoxic ^•^OH free radical production oxidative-damaging DNA. Clearly, the ESR studies showed that the PDOs bioreduction, concomitantly with the generation of the corresponding reduced analogues [14], releases ^•^OH free radical. The selective hypoxic cytotoxin PDO1 only produces this species in simulated hypoxia while the non- or poorly selective cytotoxins, PDO2 and PDO3, produce it in both conditions.

The *in vivo* results could be indicating that PDO1 and PDO2 reach the tumour on a very good concentration and after the bioreduction process drugs remain in the tumour cells. This could increase the possibility of cytotoxic events and also trigger action killing the surrounding well oxygenated cells. Future studies investigating the activity of the PDOs in combination with vitC, and with other anticancer agents *in vivo* are planned, as are other tumour models and dosing-schedules. Also a combined treatment of both compounds could be performed considering the immune response activation produces by PDO1 and the excellent size tumour reduction provides by PDO2.

##  Conflict of Interest

The authors declare that there is no conflict of interests.

## Supplementary Material

Appendix (5 pages) with supplementary data about treatment schedules (Figure 1S), determination of MTD (Table 1S), biochemical and haematological findings (Table 2S), and summary of histopathologic findings (Table 3S) is located at doi: 10.5402/2011/314209

## Figures and Tables

**Table 1:** PDO cytotoxic effects in simulated hypoxia and normoxia on different conditions and cellular systems.

Comp.	Dose (*μ*M)	SF^a,b,c^ in simulated hypoxia		SF^a,b,c^ in normoxia	
		−vitC^d^	+vitC	−vitC	+vitC
PDO2	5	3 ± 1	0 ± 0	87 ± 5	83 ± 4
PDO3	20	11 ± 2	8 ± 2	61 ± 3	74 ± 2
PDO4	5	4 ± 1	0 ± 0	67 ± 3	80 ± 5

Comp.	Dose (*μ*M)	SF_hypoxia_^a,b^		SF_oxia_^a,b^	

	20	0 ± 0		5 ± 1	MCF-7
PDO2	5	0 ± 0		34 ± 3	
	20	0 ± 0		26 ± 3	TK-10
	20	0 ± 0		18 ± 2	HT-29

^a^SF: survival fractions (%).^b^Values are means of two different experiments. The assays were done by duplicate and using at least three repetitions, and standard errors were not greater than 2 % for most assays. ^c^Using V79 cells. ^d^vitC: vitamin C (pretreatment during 25 min with equimolecular amount of vitamin).
