# Supplementary material for: Novel Phenazine 5,10-Dioxides Release •OH in Simulated Hypoxia and Induce Reduction of Tumour Volume In Vivo
Source: ISRN Pharmacol. 2011 Jun 22;2011:314209. doi: 10.5402/2011/314209 (PMC3196961; doi:10.5402/2011/314209)
Supplement: Supplementary file 1 — Appendix (5 pages) with supplementary data about treatment schedules (Figure 1S), determination of MTD (Table 1S), biochemical and haematological findings (Table 2S), and summary of histopathologic findings (Table 3S) is located at doi: 10.5402/2011/314209 [file 314209.f1.pdf]

Supplementary online material

Novel phenazine 5,10-dioxides release $^{\cdot}OH$ in simulated hypoxia and induce reduction of tumour volume in vivo

María L. Lavaggi, Mauricio Cabrera, Cristina Pintos, Carolina Arredondo, Gisela Pachón, Jorge Rodríguez, Stella Raymondo, José Pedro Pacheco, Marta Cascante, Claudio Olea-Azar, Adela López de Ceráin, Antonio Monge, Hugo Cerecetto, Mercedes González

DAY									
**1**	**2-4**	**5-7**	**8-11**	**12-14**	**15-18**	**19-21**	**22-25**	**26-28**	**29**
♦	♦	◊	♦	◊	♦	◊	♦	◊	**sacrifice**
**Begin of the treatment**									**experimental end point**

Figure 1S. Schedules of the treatment of healthy and unhealthy rats. ♦: The animals received intraperitoneally one daily dose of vehicle solution, **PDO1** (50 mg/kg b.w.) or **PDO2** (50 mg/kg b.w.); ◊: The animals rested, they did not receive any injection. All the treatments began when the tumours reached approximately 4 mm in diameter.

Table 1S. Determination of maximum tolerated dose (MTD) for drugs **PDO1**, and **PDO2**.

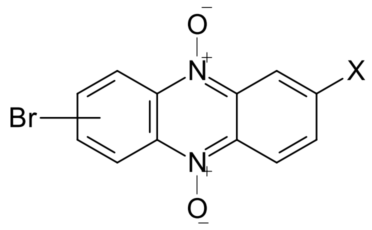

**Comp.**	**-X**	**Dose (mg/kg)**	**Weight reduction(%)[a]**
		60	3.4
**PDO2**	$-NH_2$	120	3.4
		300[b]	4.5
		60	0
**PDO1**	-OH	120	5.7
		300[b]	11.7

[a] Weight reduction is expressed as the percentage of weight reduction of treated animals respect to control animals (untreated ones) on day 3. [b] Solubility problems did not allow to study higher doses.

Table 2S. Mean values of the biochemical and the haematological findings in healthy-animals treated with **PDO1**, **PDO2**, and vehicle solution according to schedule shown in Figure 1S.

Comp.	Dose (mg/kg b.w./day)	WBC (10^9/L)[a]	HGB (g/L)[b]	HCT (%)[c]	GOT/AST (U/L)[d]	GPT/ALT (U/L)[e]	UREA (mg/dL)	CRE (mg/dL)[f]	GLU (g/L)[g]
**PDO2**	50	2.9	111	36.9	196	38	43	0.42	157
**PDO1**	50	3.9	129	41.0	158	48	40	0.44	118
**Control**	vehicle solution	1.6	99.7	30.8	319	51	42	0.46	130
**Reference values[h]**	-	6-18	110-192	35-48	47-176	35-80	15-21	0.5-1.0	50-160

[a] WBC: White blood cells. [b] HGB: haemoglobin. [c] HCT: hematocrite. [d] GOT/AST: glutamic-oxalacetate transaminase (aspartate ketoglutarate aminotransferase). [e] GPT/ALT: glutamic-pyruvate transaminase (alanine aminotransferase); normal value. [f] CRE: creatinine. [g] GLU: glycaemia. [h] From Research Animal Resources, University of Minnesota (http://www.ahc.umn.edu/rar/refvalues.html) and http://www.fauvet.fau.edu/oacm/VetData/Handouts/ratHO.htm (accessed 05/07/2010).

Table 3S. Summary of the histopathologic studies on **PDO1**-treated tumours and untreated-tumours.

OBSERVATION	PDO1-treated	untreated
Papillary cystic carcinoma	+	+
Necrosis	-	+++
Presence of immune cells	+++	+/-
Collagen	+++	+/-
Compartmentalization	+	-
Ducts and cords	-	+
